# A geological perspective on potential future sea-level rise

**DOI:** 10.1038/srep03461

**Published:** 2013-12-12

**Authors:** Eelco J. Rohling, Ivan D. Haigh, Gavin L. Foster, Andrew P. Roberts, Katharine M. Grant

**Affiliations:** 1 Research School of Earth Sciences, The Australian National University, Canberra 0200 Australia; 2 Ocean and Earth Science, University of Southampton, National Oceanography Centre, Southampton SO14 3ZH, UK

## Abstract

During ice-age cycles, continental ice volume kept pace with slow, multi-millennial scale, changes in climate forcing. Today, rapid greenhouse gas (GHG) increases have outpaced ice-volume responses, likely committing us to > 9 m of long-term sea-level rise (SLR). We portray a context of naturally precedented SLR from geological evidence, for comparison with historical observations and future projections. This context supports SLR of up to 0.9 (1.8) m by 2100 and 2.7 (5.0) m by 2200, relative to 2000, at 68% (95%) probability. Historical SLR observations and glaciological assessments track the upper 68% limit. Hence, modern change is rapid by past interglacial standards but within the range of ‘normal’ processes. The upper 95% limit offers a useful low probability/high risk value. Exceedance would require conditions without natural interglacial precedents, such as catastrophic ice-sheet collapse, or activation of major East Antarctic mass loss at sustained CO_2_ levels above 1000 ppmv.

Sea-level rise is a certain consequence of global warming. Throughout ice-age cycles of the past million years, sea levels have fluctuated between ~130 m below and ~10 m above the present level^1^,^2^,^3^. Melt-back of all ice remaining today would cause about 65 m of SLR. Modern society is vulnerable to much smaller changes in sea level^4^; some 600 million people currently live within 10 m of present-day sea level, in an area that generates 10% of the world's total GDP^5^. An assessment of 136 of the world's largest port cities estimated that, by the 2070s, the population exposed to flooding risk may grow by more than a factor of three in these cities due to the combined effects of SLR, land subsidence, population growth and urbanization, with asset exposure increasing to more than ten times current levels^6^. SLR of up to 2 m may displace almost 2.5% of the global population^7^. Understanding potential future SLR therefore is of utmost importance.

A recent expert assessment inferred a 95% probability range of 0.33–1.32 m of SLR by 2100 for 3.5°C global warming^8^. An earlier estimate, based on current ice dynamical responses^9^, inferred a total SLR of 0.8 m by 2100, with an (unlikely) maximum of 2.0 m. The lower estimate relies on two ice-contribution scenarios with realistic accelerations of outlet glacier flow speeds, whereas the high estimate was considered unlikely because it requires an instantaneous jump to high speeds.

Semi-empirical studies have established various relationships between sea level and climate parameters (e.g., temperature, forcing), and have made future projections based on IPCC emission scenarios^10^,^11^,^12^,^13^. Such studies typically project total SLR ranges between ~1 and 2 m by 2100. Other methods also suggest high future SLR values. For example, Meehl et al^14^. modelled SLR values for different emission scenarios (representative concentration pathways, RCP^15^) (Table 1). In terms of rates after 2100, the lowest RCP considered (2.6) gives an SLR rate of 0.15–0.20 m per century (m cy^−1^) (up to 1.2 m cy^−1^ at the extreme of the uncertainty envelope)^14^, while the highest RCP (8.5) gives an SLR rate of ~1 m cy^−1^ (~5 m cy^−1^ at the extreme of their uncertainty envelope)^14^. Currently observed emission rates are close to RCP8.5 (ref. 16).

Discussions of SLR and rates of SLR rapidly become abstract without sufficient context to gauge the plausibility of implicated processes and ice-retreat rates. We assess changes in the natural, pre-anthropogenic past to offer a perspective of what nature has done before and so might do again. Our assessment is guided by two questions. First: is today's climate system in equilibrium with forcing? Second: what are the natural timescales and rates of change in ice-volume adjustment to a disequilibrium state, relative to a forcing increase? We first assess geological data for (approximate) answers to these questions, and then use the compiled information in a probabilistic evaluation of the natural context to future SLR projections. Because we assess a wide range of SLR rates and adjustment timescales from available geological evidence, we implicitly consider a natural range of forcing types (orbital, major feedbacks) and rates (up to 1.5 W m^−2^ ky^−1^ of annual mean global forcing, or about 3 W m^−2^ ky^−1^ of annual mean forcing at high northern latitudes^17^, where ky stands for thousand years). Anthropogenic rates of climate forcing, close to a global mean of 1 W m^−2^ cy^−1^, are much faster than the natural rates considered. For our forward look we assume that – at least – the modern forcing by CO_2_ levels of 392–394 ppmv is maintained (no reduction). Our aim is to portray the plausible longer-term consequences of naturally precedented SLR rates and adjustment timescales. We then use that context to assess whether modern observed responses are exceptional/unique to the industrial period.

## Results

The answer to the first question, whether today's climate system is in equilibrium with forcing, is negative. Anthropogenic climate forcing is more than an order of magnitude faster than climate forcing or major feedbacks at any known time in the Cenozoic^18^. Key climate-system components, such as deep ocean temperature and ice volume, respond slowly due to their large inertia. Ice-volume contributions to future SLR will therefore reflect delayed responses to GHG emissions, developing climate system feedbacks, and future emissions. The large and fast-growing disequilibrium between accelerated climate forcing and slow/lagging response thus creates a strong potential for rapid sea-level adjustments. The current disequilibrium may be evaluated by comparing present-day conditions with geological data that illustrate the likely climate-system state if it had been given sufficient time to respond completely to the change in forcing.

**Figure 1:**
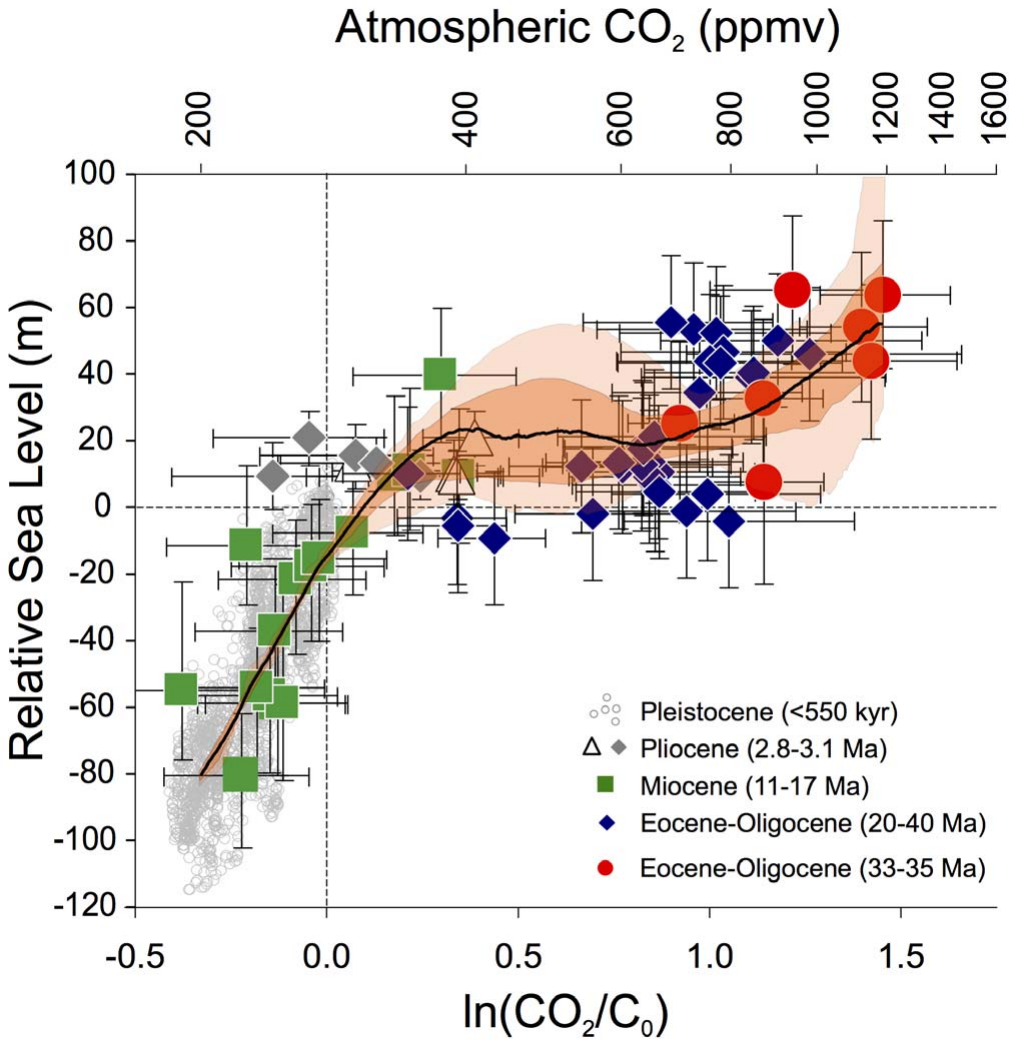
Sea-level versus CO_2_ concentrations (and the logarithmic radiative forcing influence of CO_2_ changes^17^,^28^, expressed by ln(CO_2_/C_0_), where C_0_ represents the preindustrial CO_2_ level of 278 ppmv), after ref. 24. Symbols represent reconstructions with uncertainties for different intervals of the past 40 million years. The black line and orange envelopes represent a probabilistic assessment that takes into account full propagation of all uncertainties (black line is the probability maximum; dark orange is the 68% probability interval; light orange is the 95% probability interval)^24^. The relationship averages over orbital configurations. Hence, at any given CO_2_ concentration, periods with ‘warmer/colder than average’ orbital configurations for the northern hemisphere may have had higher/lower sea level, respectively (e.g., Last Interglacial sea level reached 8–9 m above Holocene values, although CO_2_ concentrations were similar).

Some studies have evaluated the natural relationship between climate change and sea-level response^e.g.^^19^,^20^, but continuous, high-resolution sea-level records are needed for a sound observation-based assessment. Rohling et al^2^. presented such a record, and quantified the relationship between sea level and polar temperature^21^ over the last 500,000 years. They assumed a 2:1 ratio between Antarctic and global mean temperature variability (estimates range from 1.2 to 2.5; refs. 17,22,23) and their dataset for the last 500,000 years was dominated by climates colder than today. To better extrapolate into warmer states, Foster and Rohling^24^ compiled results from periods both warmer and colder than today, during the past 40 million years (Figure 1). They avoided complications involved in calibrating Antarctic or deep-sea temperature data to global mean temperature, by instead using CO_2_ reconstructions to compare with sea-level reconstructions. Their dataset includes periods of cooling/CO_2_ decrease and warming/CO_2_ increase. For CO_2_ levels below 600 ppmv, these trajectories appeared indistinguishable, which instils confidence that the CO_2_:sea-level relationship provides useful information about natural longer-term responses expected for anthropogenic CO_2_ increases.

The inferred CO_2_:sea-level relationship is non-linear (Figure 1). Below ~350 ppmv, sea level increases almost linearly with increasing CO_2_. The curve then flattens until CO_2_ reaches 700 ppmv, above which sea level rises strongly again with CO_2_ increase. The ‘plateau’ for CO_2_ between ~350 and 700 ppmv, with sea level within a range of 22^+13^/_−12_ m above present, likely represents a climate state in which the (relatively sensitive) ice sheets of Greenland, West Antarctica, and marine-based parts of East Antarctica were severely reduced or eliminated^17^,^20^. The large East Antarctic ice sheet (EAIS) is more stable, and only contributes significantly to sea-level change when CO_2_ is above 700 ppmv. This agrees with modelling of CO_2_ sensitivity of the EAIS, which suggests a ~700 ppmv threshold^25^,^26^. Note that the degree of hysteresis in EAIS growth and decay remains debated; CO_2_ may need to rise above 1000 ppmv before EAIS contributions to SLR become relevant^26^,^27^.

Annual mean CO_2_ levels reached 392–394 ppmv in 2011–2012. If this level is maintained, then the sea level:CO_2_ relationship^24^ suggests a natural longer-term climate state with equilibrium sea level at 24^+7^/_−15_ m above the present level (68% probability). This raises our second question, which concerns the timescales needed for such sea-level adjustments. To answer it, we need information about rates of SLR.

It is less straightforward to use geological data to answer the second question because present-day climate change due to rapid GHG emissions is: (*a*) unprecedentedly rapid^18^ relative to changes due to orbital forcing and climate system feedbacks^17^,^28^, and (*b*) becoming warmer than a normal interglacial^29^. Regardless, geological observations can at least provide a sound natural context for modern trends and future projections. Highly resolved sea-level data, as required to quantify rates of SLR, span the five most recent ice-age cycles (~500 ky) (refs. 2,30,31).

Information about rates of SLR is most easily obtained from deglaciations, when ice ages terminated and sea level rose by up to 120–130 m at mean rates of about 1 m cy^−1^, but with rapid steps bracketed by slower episodes^31^,^32^,^33^,^34^,^35^,^36^,^37^. During one of these rapid steps (‘meltwater pulse 1a; mwp-1a’), SLR rates reached 4–5 m cy^−1^ for several centuries^36^. Rapid steps of > 2 m cy^−1^ also occurred during previous deglaciations^2^,^30^,^31^,^35^. Note that past deglacial SLR rates characterise transitions from glacials with 2–3 times the present-day ice volume, to interglacials with ice volumes similar to the present.

Away from deglaciations, data for 75–30 ky ago, when sea level fluctuated between about 60 and 90 m below the present level, reveal rates of SLR when major Northern Hemisphere ice sheets were consistently present (with fluctuating volume). This is a significantly different state than deglaciations. Grant et al^31^. provided improved age control and uncertainty propagation relative to earlier quantifications^38^,^39^,^40^, which revealed that all phases of considerable ice-volume reduction had SLR rates of 1–2 m cy^−1^ (comparable with mean rates during deglaciations). This suggests that peak rates during deglaciations may reflect special conditions, but that rates of 1–2 m cy^−1^ are not exceptional for natural fluctuations. Nevertheless, these rates concern times with much greater ice volume than today, and with intense global climate fluctuations^17^,^28^,^31^,^41^,^42^,^43^,^44^.

The most valuable information on rates of SLR comes from periods when global ice volumes were similar to present. The last five glacial cycles contain two interglacials that were up to 2°C (ref. 45) warmer than the pre-industrial state, with sea level up to 10 m higher than today^2^,^3^,^30^,^46^,^47^,^48^. Few data cover the oldest of these, centred at around 404 ky ago, but the Last Interglacial (LIg; ~130–115 ky ago^48^) has been extensively studied. LIg global temperature was about 1 ± 0.5°C higher than pre-industrial temperature^45^ and sea level peaked 6–9 m above the present level^46^,^47^,^48^, which implies a 10–15% ice-volume reduction relative to present. Initial (Red Sea-based) LIg SLR rate estimates of 1.6 ± 1.0 m cy^−1^ lacked direct age control^46^. Subsequent studies proposed 1000-year average LIg rates of > 0.26 m cy^−1^ (ref. 49) and 0.56–0.92 m cy^−1^ (ref. 47), which is consistent with a 1000-year smoothed estimate of 0.7 ± 0.4 m cy^−1^ over the −5 to +5 m sea-level range based on improved dating of the Red Sea record^31^. Note that such smoothing masks brief intervals with more rapid rise. Data from western Australia suggest a rapid rise within the LIg at 0.6 m cy^−1^ (ref. 50). We infer that LIg SLR likely occurred at sustained rates of ~1 m cy^−1^ or less.

**Figure 2:**
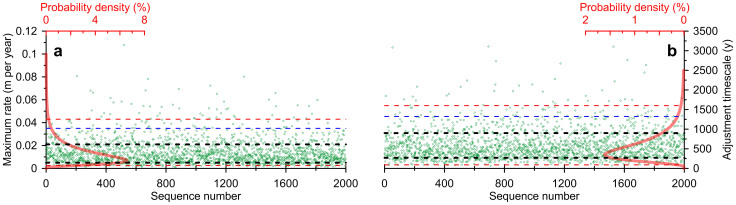
Probability distributions (red) for: (a). maximum rates of SLR (shown in m y^−1^, as used in the calculations); and (b). adjustment timescales (in y), as discussed in the text. The green dots indicate the 2000 random samplings of the probability distributions for our probabilistic assessment of natural sea-level change based on equations 1–3. Dashed red lines indicate the 2.5^th^ and 97.5^th^ percentiles that delimit 95% probability intervals; dashed blue lines indicate 95^th^ percentiles that give the upper bound of the 90% probability intervals (to avoid clutter the lower bound has been omitted); and dashed black lines indicate 16^th^ and 84^th^ percentiles that delimit the 68% probability intervals.

**Figure 3:**
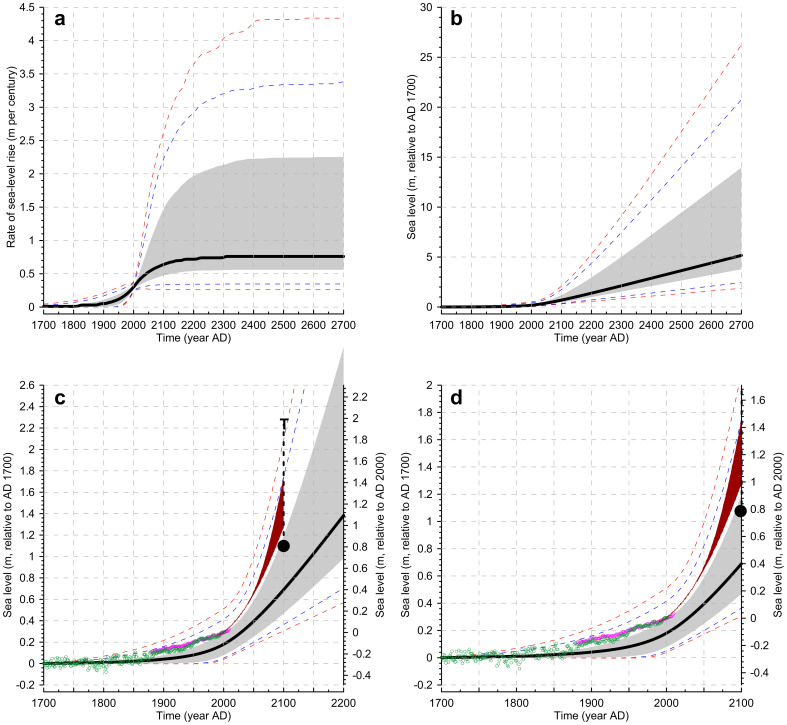
Probabilistic assessment of natural sea-level change based on equations 1–3. The heavy line is the probability maximum (peak of the probability distribution), the grey envelope marks the 68% probability interval, and the dashed blue (red) lines mark the 90% (95%) probability intervals, respectively. (a). Rates of SLR relative to 1700, in m cy^−1^ (i.e., 100 × result from equation 1). (b). SLR after equation (2). (c). Zoomed-in portion of (b), ending at 2200. The brown wedge is the range of semi-empirical projections by Vermeer and Rahmstorf^12^, the heavy dot outlines the most-likely projection by Pfeffer et al^9^., and the heavy dashed black line represents the full range of SLR estimates of Pfeffer et al^9^. Historical sea-level reconstructions of Jevrejeva et al^11^. (green dots) and Church and White^51^ (magenta dots) are also shown. (d). As (c), but zoomed in on 1700–2100.

Here, we capture the (above) compiled geological observations of past rates, and also of timescales, of ice-volume/sea-level adjustment in broadly defined probability distributions (*Methods*; Figure 2). We then develop a probabilistic assessment of SLR, and use this natural context to discuss historical SLR trends and future projections (*Methods*, Figure 3).

## Discussion

Within the wide parameter ranges considered (*Methods*; Figure 2), previous high estimates of about 0.8 to 1.3 m SLR by 2100 refs. 8,9, and historical SLR trends^11^,^51^, fall near the upper bound of our 68% probability interval of natural (pre-anthropogenic) change (Figures 3c,d). Semi-empirical SLR projections^12^ (Figure 3c,d) accelerate in high-emission scenarios to high SLR values from about 2050, but in low-emission scenarios remain close to the upper bound of our 68% probability interval.

The lower half of our 68% probability interval relates to input parameter values that are close to LIg values (see above). Coincidence of recent observations and projections with the upper bound of our 68% probability interval therefore suggests that current SLR responses are high by a natural interglacial standard. However, coincidence with the 0.8 m SLR estimate for 2100 based on ice-dynamics^9^ suggests that tracking the upper bound of our 68% probability interval does not require unprecedented processes. Here, we note that this 0.8 m estimate^9^ is based on extrapolation of well-constrained processes for Greenland to a global context, but that proportionally greater contributions might be possible from Antarctica, where marine grounded channels are not well-defined. We also emphasise that, given the multi-centennial timescales of full adjustment (*Methods*), 0.8 m by 2100 represents a transient mean for the current century along a trajectory toward rates of almost 2 m cy^−1^ by about 2200 (Figure 3a). LIg data suggest that rapid rates may be sustained at least until SLR reaches +8 to +10 m (i.e., until ~2500 for the upper bound of our 68% interval; Figure 3b).

SLR in semi-empirical projections for high emission scenarios reaches toward the upper bound of our 90% interval (Figure 3c,d). This therefore requires development of: (1) ice-loss processes without known precedents in past warm (interglacial) intervals, leading to eventual rates of SLR of almost 3.5 m cy^−1^ (Figure 3a), and (2) unexpected increases to modern outlet-glacier flow regimes, relative to the assessment of Pfeffer et al^9^. This does not mean that such scenarios cannot occur; we only infer from the natural context that such potential developments would be unique to the anthropogenic era.

Given the need for long-term planning in coastal defence, we consider a ‘worst case’ outlook from our natural perspective. The upper bound of our 95% probability envelope (i.e., the 97.5^th^ percentile) implies a 2.5% chance of > 1.8 m SLR by 2100 and > 5.0 m by 2200, relative to 2000 (Figure 3). This 2100 value closely approaches the (unlikely) maximum value suggested by Pfeffer et al^9^. (Figure 3c). However, this trajectory requires that SLR rates develop toward an eventual value of 4.3 m cy^−1^, roughly similar to mwp-1a (ref. 36) (Figure 3a), even though today's global ice volume is only about a third of that at the onset of the last deglaciation. Most of the extra ice during glacial times existed in North America and northwestern Eurasia, where it extended to relatively low latitudes (40–50°N). These ice sheets were highly sensitive to climate change, as witnessed by the fact that they existed during ice ages and were almost entirely absent during interglacials. Both the size and sensitivity of these glacial ice masses would have been conducive to high deglacial rates of SLR. Starting from present-day conditions, rates such as those of mwp-1a would require unprecedented ice-loss mechanisms, such as collapse of a major ice sheet (e.g., the largely marine-based West Antarctic Ice Sheet). Alternatively, such rates might develop with a large increase in the amount of ‘vulnerable’ ice, by activation of major EAIS retreat. From the natural perspective, however, the latter only seems to become relevant under extreme GHG forcing, with long-term CO_2_ above ~1000 ppmv or so (see Figure 1, and discussion above). Without invoking such exceptional conditions or catastrophic events, our assessment supports the notion^9^ that ~2 m of SLR by 2100 represents a useful upper limit. Improved (less smoothed, and globally documented) SLR rate estimates from past interglacials are needed to further refine this value, and especially the risk of massive ice-sheet collapse.

Early historical SLR data are scattered because there are few long tide-gauge records, but from ~1880 the record is well defined (Figure 3c,d). While the data generally coincide with the upper half of our 68% probability interval for naturally precedented changes, the observations contain some superimposed multi-decadal variability, for which there are several explanations. Since the 1950s, increased water storage on land behind man-made dams has led to reduced SLR^52^, and increased volcanic eruptions in the latter half of the 20^th^ century increased atmospheric albedo and reduced ocean heat content, limiting thermal expansion^53^. In addition, relatively rapid initial SLR may have arisen because proportionally more heat remained in the ocean – where it rapidly caused thermal expansion – while less heat was used for (slower) ice-sheet retreat. Since 1950, this balance may have shifted to reducing contributions from thermal expansion and accelerating ice-volume contributions. The latter interpretation is consistent with observations of recent increasing mass loss from major ice sheets^54^,^55^,^56^. Our evaluations are not designed to reproduce such multidecadal variability, but in a sensitivity test we have included representative annual, interannual, and multidecadal variability of differing amplitudes into our 2000 iterations of SLR rates (Supplementary Information Figures S1,S2). This results in only marginal widening of the probability intervals relative to Figure 3, and our conclusions are not affected.

Overall, the most important point from our assessment is that SLR since 1700 has been consistent (within uncertainties) with expectations from geologically documented responses to climate-system disequilibria. Thus, no matter how special anthropogenic climate forcing may be in terms of magnitude and rate of increase, the observed sea-level response to anthropogenic forcing has so far remained close to the range of expectations based on well-known natural precedents.

Continued monitoring of SLR, and comparison with the natural context outlined here, may be used to identify if and when sea-level response becomes ‘special’ (i.e., unprecedented during geological interglacials). For example, the higher emissions projections of Vermeer and Rahmstorf^12^ imply a shift toward such ‘special’ responses from ~2050. Until such time, however, comparison of SLR observations with our results indicates that future SLR projections can rely with confidence on testing and validation of physical models against well-known examples from the recent geological past.

## Methods

The disequilibrium between rapidly increased anthropogenic climate forcing and slow ice-volume responses amounts to many metres of SLR at current atmospheric CO_2_ levels, and may not change significantly with increasing CO_2_ to 700 ppmv (Figure 1)^24^. Temperature adjustments to emission scenarios within that range follow a sigmoidal pattern with time, accelerating and then decelerating, with adjustment timescales between 150 years for low-emission scenarios and 400 years for high-emission scenarios^15^. We infer that the present disequilibrium is already sufficient to cause build-up toward major ice-sheet responses, and that further warming will occur over similar timescales as the developing ice-sheet responses, so that increased forcing would cause (rapid) shifts toward extremes of the parameter ranges considered here (high ultimate SLR rates and rapid adjustment timescales). The median global radiative forcing projections for high-emission RCPs is ~12 W m^−2^ (ref. 15). For deglaciations, this was 8–10 W m^−2^ (refs. 17,28). We infer that the long-term consequences of high-emission RCPs may be suitably gauged from the SLR adjustment rates and timescales of deglaciations, if we assume that such consequences would develop via naturally precedented processes (e.g., because the response becomes rate-limited). Similar to a previous study^57^, we consider that the long-term consequences of low- to middle-emission RCPs may be gauged using LIg responses. We proceed on this basis to formulate our ‘natural context’.

**Figure 4:**
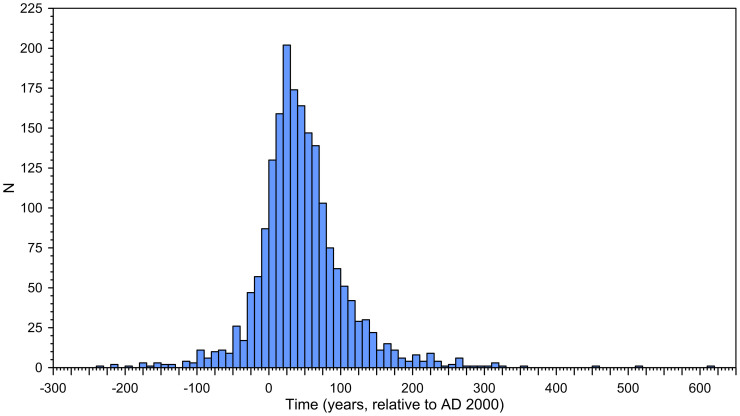
Histogram of *t_inf_*, the relative age of the inflection from concave to convex in our 2000 sigmoidal (logistic) SLR rate reconstructions.

The processes that govern mass-loss from ice sheets build up gradually because of dynamical spin-up or due to slow increases in early melting until ice-sheet height-temperature feedbacks lead to rapid acceleration^9^,^54^,^58^,^59^. Hence, our assessment allows rates of SLR to gradually build, and then accelerate, before settling at the maximum achievable rate for the mechanisms involved (α in our analysis below, in m y^−1^). We approximate this using a logistic function of the form: 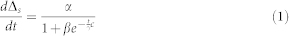
Here γ is the timescale over which the rate increases from zero to its peak value (in y). β is discussed below. The dimensionless scaling constant *C* is empirically set so that γ equals the period over which *d*^2^*Δ_S_*/*dt*^2^ exceeds 1% of its maximum value. *C* is independent of α, β, and γ; it depends merely on the cutoff criterion used for *d*^2^*Δ_S_*/*dt*^2^. Our consistently applied criterion implies that *C* = 12 in all iterations. The timing of the central inflection in *dΔ_S_*/*dt*, the switch-over from accelerating to decelerating rates of SLR (with the constraint that at AD2000, the SLR rate is 3 ± 1 mm y^−1^, see below), is *t_inf_* = γ ln(β)/*C*. A histogram for all 2000 iterations (Figure 4) suggests that *t_inf_* falls within a window of ~300 years. For comparison, modeled developments from zero to maximum SLR rates from Greenland, over a wide range of climate forcing scenarios, suggest inflection-point timings within a 200-year window (2100–2300) (analysis based on Figure 3b of ref. 59), which is qualitatively similar to our inference.

Integration of equation (1) gives *Δ_S_* (i.e., SLR) in m relative to pre-industrial times (AD1700): 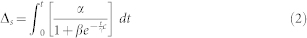
All calculations are performed in m y^−1^, but we present results in m cy^−1^ for comparison with previous studies. The dimensionless parameter β is set so that the rate of SLR at AD2000 amounts to 3 mm y^−1^ (i.e., 0.003 m y^−1^ or 0.3 m cy^−1^), with a Gaussian uncertainty distribution over a range of ± 1 mm y^−1^ (represented by *rnd*(0.001) in equation 3), based on observations from satellite altimetry for 1993 to present^51^: 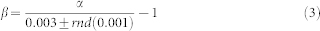
Geological data constrain a broad probability distribution for α. Rates must be > 0 m cy^−1^ for SLR to occur. The probability peak may be expected at around the rate of LIg SLR above present levels of 0.56–0.92 m cy^−1^ or 0.7 ± 0.4 m cy^−1^ (refs. 31,47,50) (i.e., < 1.1 m cy^−1^), with the caveat that 1000-year averaged LIg estimates may mask shorter episodes with higher values. Mwp-1a, with rates of SLR up to ~5 m cy^−1^ (ref. 36), provides a real-world example of naturally occurring extreme rates. Accordingly, we formulate a simple lognormal distribution, so that α > 0 m cy^−1^, with 50% of possibilities for α ≤ 1.1 m cy^−1^, and with 99% of α ≤ 5.0 m cy^−1^ (red curve in Figure 2a).

Similar conditions exist for the adjustment timescale (γ). The lower constraint must be γ > 0 y. Where detailed records exist, rates of SLR tracked rates of polar temperature change within a few centuries, while sea-levels tracked a new temperature stasis within 2 to 7 centuries^31^. However, response times of about a millennium or more may not be excluded^59^,^60^. For example, mass-loss rates of the Greenland ice-sheet develop toward maxima over about 750 years in a recent model^59^. Hence, we define a lognormal distribution so that γ > 0 y, with 50% of possibilities for γ ≤ 500 y, and with 99% of γ ≤ 2000 y (red curve in Figure 2b).

Equations 1–3 are solved in a probabilistic assessment. In a Monte-Carlo style approach, we performed 2000 independent random samplings of the α and γ distributions (Figure 2), and of β (equation 3). Next, we evaluated per annual time-step the probability distribution defined by the 2000 solutions (95% probability limits using the 2.5^th^ and 97.5^th^ percentiles, 90% limits with the 5^th^ and 95^th^ percentiles, and 68% limits using the 16^th^ and 84^th^ percentiles). Results are shown in m cy^−1^ in Figure 3a, and those for SLR in m (eq. 2) in Figure 3b–d.

## Author Contributions

E.J.R. led the analyses and writing of the manuscript. All authors added through discussions and reviews of the study, especially A.P.R. and K.M.G. over the past few years of our Red Sea sea-level collaboration. G.L.F. provided a deep-time geological perspective and added Figure 1. I.D.H. added focus on modern implications and assisted with the statistical assessment.

## Supplementary Material

Supplementary Information Supplementary Information

## Figures and Tables

**Table 1:** Recent projections^14^,^15^ of CO_2_ levels, global warming, and SLR

RCP	CO_2_ (ppmv)	Warming by 2100 (°C)	SLR by 2100 (m)	Warming by 2200 (°C)	SLR by 2200 (m)
2.6	Peak at 440, Stabilised at 360	~1.8	0.25	~1.5	0.39
4.5	Stabilised at 540	~2.6	0.37	~3.0	0.71
8.5	2000+	~5	0.56	>7	1.53

RCP = Representative Concentration Pathways.
