# Supplementary material for: A geological perspective on potential future sea-level rise
Source: Sci Rep. 2013 Dec 12;3:3461. doi: 10.1038/srep03461 (PMC3860070; doi:10.1038/srep03461)
Supplement: Supplementary Information [file srep03461-s1.pdf]

Supplementary information to:

A geological perspective on potential future sea-level rise

Eelco J. Rohling, Ivan D. Haigh, Gavin L. Foster, Andrew P. Roberts, and Katharine M. Grant

This Supplementary Information shows results from our sensitivity tests. We performed the same experiments as discussed in the main text, but superimposed additive (over a range of +/- 0.001 m/y, independent of SLR rate) as well as multiplicative (randomly varying the multiplication rate over a range of 0.8 to 1.2 times the SLR rates from the underlying sigmoidal component) elements of shorter-term variability/noise. These elements contain both sinusoidal multidecadal and interannual components with randomly generated phase and amplitude, and randomly generated annual noise. Representative examples of the records with superimposed shorter-term variability/noise are shown in Figure S1. Clearly, such shorter-term components can considerably advance or delay the inflection time of the underlying sigmoidal (logistic) component, while signal amplitudes vary strongly between records, reflecting realistic uncertainties.

As illustrated in Figure S2, addition of these shorter-term components of variability/noise marginally widens the probability intervals shown in main-text Figure 3. However, none of the conclusions reached in our study are affected by this. We conclude that our results are robust relative to the assumption of only a smooth, long-term pattern of change.

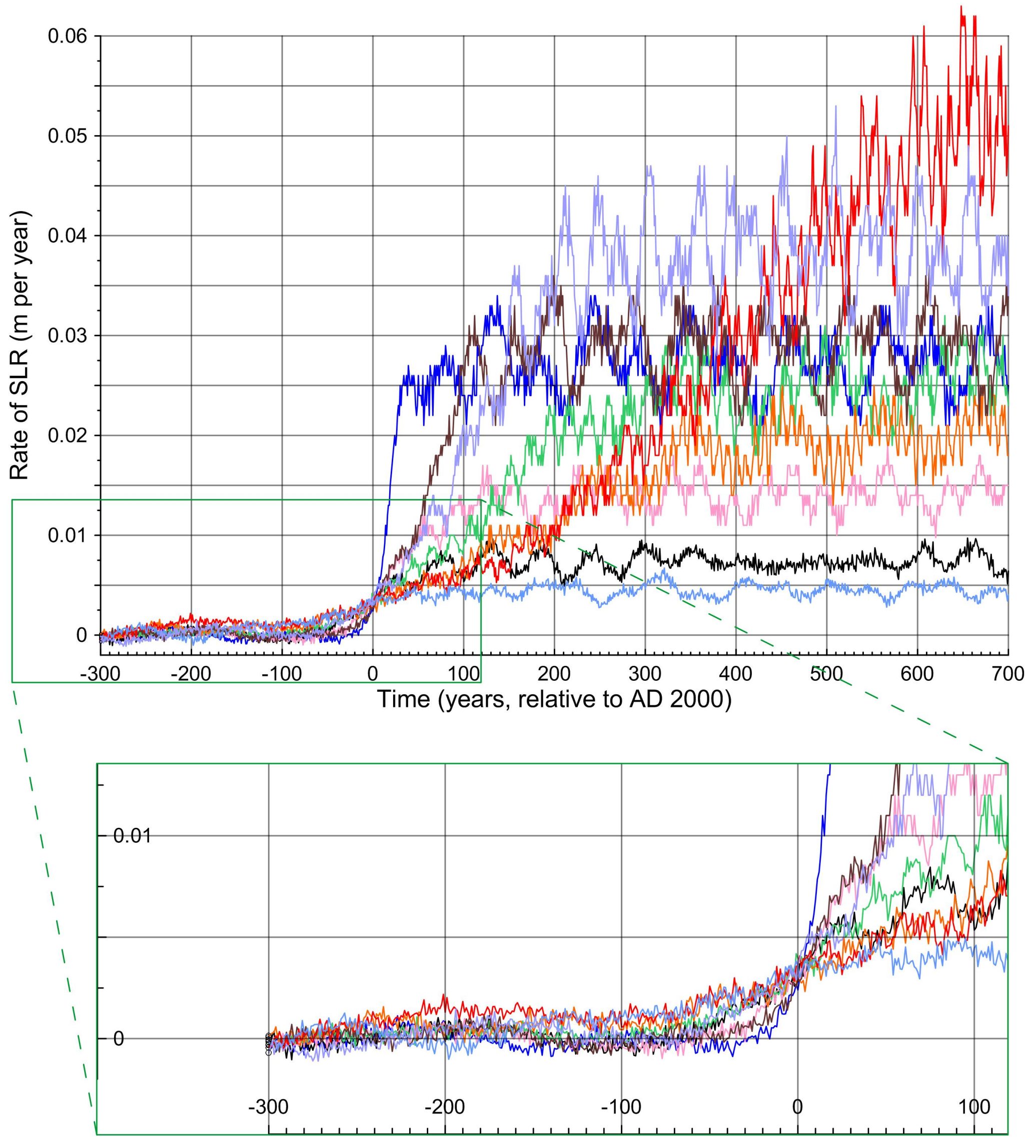

Figure S1. Representative examples of 2000 SLR rate records developed within the parameter ranges discussed in *Methods*, but including superimposed shorter-term variability/noise elements. The inset illustrates details of the early parts of the records and their passing through an AD 2000 value of 0.003±0.001 m y^{-1}. Note that these records are shown in m y^{-1} (as per the calculations), which in Figure S2 is converted into m cy^{-1} for ease of comparison with other studies.

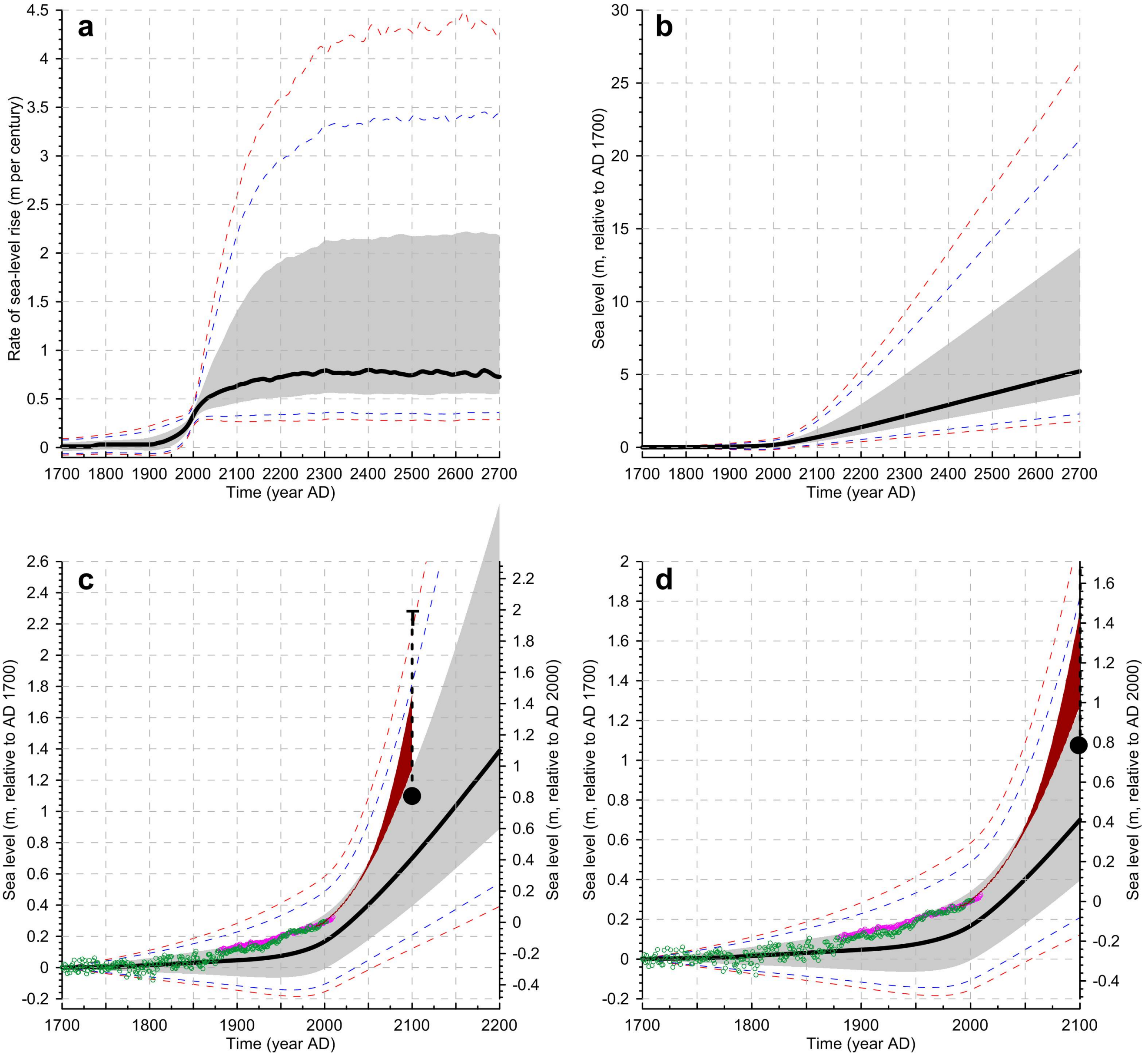

Figure S2. As main-text Figure 3, but including superimposed variability/noise (see examples in Figure S1).
